# Differential P-Glycoprotein/CD31 Expression as Markers of Vascular Co-Option in Primary Central Nervous System Tumors

**DOI:** 10.3390/diagnostics12123120

**Published:** 2022-12-10

**Authors:** Tiziana Annese, Mariella Errede, Antonio d’Amati, Michelina De Giorgis, Loredana Lorusso, Roberto Tamma, Domenico Ribatti

**Affiliations:** 1 Department of Medicine and Surgery, LUM University, Casamassima, 70100 Bari, Italy; 2 Department of Basic Medical Sciences, Neurosciences and Sensory Organs, University of Bari Medical School, 70124 Bari, Italy; 3 Section of Pathology, Department of Emergency and Organ Transplantation, University of Bari, 70124 Bari, Italy

**Keywords:** angiogenesis, anti-angiogenic therapeutics, brain tumors, double immunohistochemical staining, drug resistance, P-glycoprotein, tumor progression, vascular co-option

## Abstract

Background: Vascular co-option is one of the main features of brain tumor progression. It is identified using histopathological analysis, but no antibody-specific markers were found, and no universally accepted histological features were defined. Methods: We employed double immunohistochemical stainings for CD31, P-gp, S100A10, and mitochondria on formalin-fixed, paraffin-embedded human samples of IDH-WT glioblastoma, IDH-mutant astrocytoma, and meningioma to study vascular co-option across different brain tumors and across normal, peritumoral, and intratumoral areas using the Aperio colocalization algorithm, which is a valid and robust method to handle and investigate large data sets. Results: The results have shown that (i) co-opted vessels could be recognized by the presence of metabolically overactive (evaluated as mitochondria expression) and P-gp^+^ or S100A10^+^ tumor cells surrounding CD31^+^ endothelial cells; (ii) vascular co-option occurs in the intratumoral area of meningioma and astrocytoma; and (iii) vascular co-option is prevalent in peritumoral glioblastoma area. Conclusions: The described approach identifies new markers for cellular components of the vessel wall and techniques that uncover the order and localization of vascularization mechanisms, which may contribute to developing new and possibly more effective therapeutic strategies.

## 1. Introduction

Brain tumors are a wide and heterogeneous group of rare neoplasms even though their incidence is increasing worldwide. Among this group, glioblastoma is one of the most common and deadliest brain tumors. In fact, even with the introduction of optimized standard treatments, including extensive surgical resection, radio-chemotherapy, tumor treating fields (TTFs), and anti-angiogenic therapies (AATs), the overall survival did not show a significant improvement and currently remains very limited [1,2].

Glioblastoma is a highly vascularized and hypoxic tumor characterized by overexpression of pro-angiogenic factors. In glioblastoma as well as in many other malignant neoplasms, AATs have been employed to disrupt pre-existing blood vessels, also acting as adjuvants to normalize abnormal vasculature [3,4]. Gliomas use various neovascularization mechanisms, which may coexist and cooperate for tumor growth and progression [5,6], including vascular co-option [7], angiogenesis [8], intussusceptive microvascular growth [9], vasculogenesis [10,11,12], vascular mimicry [3,13], and glioblastoma-endothelial cell trans-differentiation [14]. 

Vascular co-option is defined as “the establishment of a stable anatomical and/or functional interaction between the co-opting cell and the co-opted blood vessels” [15]. This mechanism is used by tumor cells as an adaptive strategy to hijack host endothelium vessels and have direct access to oxygen and nutrients, consequently increasing their growth and invasive potential. 

Vascular co-option is employed by various tumors, including primary and metastatic brain tumors, to spread into the host tissue or colonize new tissues by extravasation, respectively [16,17].

Functional experiments using in vivo models have demonstrated that the co-option of pre-existing capillary networks is a critical step for the initiation of multiorgan metastases from different cancer types. Different cell types have been demonstrated to interact with pre-existing vessels. The process of vascular co-option mediates their developmental program, favors their survival, maintains their self-renewal capacity, and could influence the emergence of cell heterogeneity by inducing specific functional properties to co-opting cells [17].

As demonstrated in experimental studies, vascular co-option appears to be the first mechanism in the temporal sequence of the gliomas vascularization process due to the characteristic angiotropism of neoplastic cells, which establish an intimate contact with normal brain microvessels, especially at the invasive border of the tumor [6,8,18].

Vascular co-option is identified using histopathological analysis, but no marker adequately distinguishes co-opted blood vessels from angiogenic ones [19,20,21]. P-glycoprotein (P-gp), also known as ATP-dependent translocase ABCB1 or CD243 or MDR-1, is a drug efflux ABC transporter present on the cell membrane of normal cells, such as endothelial cells (ECs) and tumor cells [22]. P-gp acts as a drug translocator across the membrane to avoid drug accumulation in multidrug-resistant cells [23,24,25], and it is a flippase [26] because it flips the phospholipids from the cytoplasmic to the exoplasmic side of the apical membrane [23]. ECs of the intact blood–brain barrier (BBB) normally express P-gp, but its expression is reduced in case of BBB breakdown, as observable in hypoxic and necrotic parts of glioblastoma multiforme [27,28]. P-gp is also expressed in tumor cells and contributes to chemotherapy resistance, reducing drug bioavailability and accumulation around microvessels [29,30]. Because ECs typically express P-gp, also during fetal brain development [31], its expression by tumor-associated ECs may have a limited role in multi-drug resistance (MDR). In contrast, tumor-associated perivascular astrocytes and glioma cells contribute to the MDR profile of tumoral vessels in a more significant manner [27].

S100 is a group of proteins controlling different biological activities [32] associated with tumor microenvironment regulation and glioma prognosis [32]. A member of this family, S100A10, is a specific biomarker of differentiated non-stem glioma cells [33] mediating cell–cell contact among glioma cells and ECs [34]. Moreover, S100A10 may form with ANXA2, a heterotetramer, acting as a plasminogen receptor and correlated with tumor aggressiveness and progression [35,36]. 

**Figure 1:**
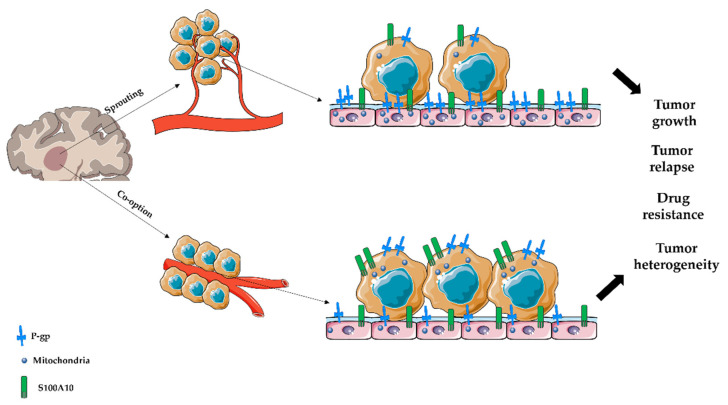
P-gp, S100A10, and mitochondria expression by tumor cells or ECs during sprouting angiogenesis or vascular co-option. Based on our observation and the literature, we postulated that where sprouting angiogenesis occurs: tumor cells can be identified as P-gp^+^ or S100A10^+^ cells with a moderate proliferating profile (evaluated as mitochondria positivity); ECs overexpress P-gp and mitochondria compared with tumor cells and with ECs in the area where vascular co-option occurs. Moreover, we postulated that where vascular co-option occurs: tumor cells overexpress P-gp, mitochondria, and S100A10 compared with ECs and with the tumor cells in the area where sprouting angiogenesis occurs; ECs express P-gp, mitochondria, and S100A10 but lesser than tumor cells or the ECs in the area where sprouting angiogenesis occurs. These different ways of tumor progression could mainly involve diverse mechanisms such as tumor growth, heterogeneity and relapse, and drug resistance.

As well explained elsewhere [37], here, we employ double immunohistochemical stainings for CD31, P-gp, S100A10, and mitochondria (as a marker of active aerobic metabolism) on formalin-fixed, paraffin-embedded human samples of IDH-WT glioblastoma, IDH-mutant astrocytoma, and meningioma to study vascular co-option using the Aperio colocalization algorithm (Leica Biosystems, Nussloch, Germany), which is a valid and robust method to handle and investigate large data sets. In vascular co-option areas, we expected to observe P-gp- or S100A10-positive tumor cells surrounding blood vessels with reduced CD31, P-gp, and mitochondria expression. On the contrary, in the areas of sprouting angiogenesis, we expected the blood vessels to show an increased CD31 expression, P-gp, and mitochondria expression, which are indicative of ECs proliferation and migration. Furthermore, we compared the findings between three different CNS tumors: glioblastoma IDH-WT, astrocytoma IDH-mutant, and meningioma (Figure 1).

IDH-WT glioblastoma is an aggressive and diffuse astrocytic glioma that lacks mutations in IDH1, IDH2, and histone H3 genes. Histologically, it is characterized by hypercellularity, high mitotic index, microvascular proliferation, and necrosis. All of these morphological features, along with the absence of IDH and H3 mutations, are essential criteria for diagnosing IDH-WT glioblastoma. IDH-WT glioblastoma is always graded 4, according to the WHO 5th Classification of CNS Tumors.

IDH-mutant astrocytoma IDH-mutant is a diffuse astrocytic glioma, presenting mutations in IDH1 or, less frequently, IDH2 genes. Histologically, it is characterized by atypical and infiltrating glial cells. The astrocytoma grading may vary from grade 2 to grade 4 depending on the presence of high mitotic index, microvascular proliferation, and necrosis. 

Meningioma is the most common primary CNS tumor that arises from arachnoid cap cells of the dura mater. Histologically, it is characterized by the presence of elongated cells with frequent nuclear pseudo-inclusions arranged in fascicles and whorls. Many histological types have been described for meningiomas, and some are related to a higher risk of recurrence and aggressiveness. Based on specific criteria described in the WHO 5th Classification of CNS Tumors, meningioma grading may vary from grade 1 to 3 [38]. Among these criteria, the most relevant is represented by the number of mitosis per 10 high-power fields. Vascular proliferation is not included in meningioma grading criteria even though previous studies demonstrated the critical role of angiogenesis and vascularization in this tumor [39].

In our study, we used an immunohistochemical approach in order to identify the presence of vascular co-option and to analyze any possible difference occurring between these three different CNS tumors.

## 2. Materials and Methods

### 2.1. Patients and Ethical Approval

The present study was carried out on biopsies of primary central nervous system tumors (n = 30 IDH-WT glioblastomas; n = 25 IDH-mutant astrocytomas; n = 22 meningiomas) obtained from patients who underwent surgery before any therapy and were diagnosed at the Pathology section of the University of Bari (Table 1).

The study was reviewed and approved by local ethics committee guidelines following the Helsinki Declaration of 1975. All patients gave signed informed consent for diagnostic and research analyses.

The histological diagnosis was determined according to the 5th Central Nervous System Tumors Classification of the World Health Organization [38]. Furthermore, clinical data and histological features such as cytological atypia, mitotic activity, endothelial cell proliferation, and necrosis have been evaluated to assess tumor grade. 

Tissues were collected at surgery time, fixed in 10% buffered formalin for 16–24 h, and temporarily stored in 70% ethanol until processing. After dehydration in graded ethanol and clarification in xylene, biopsies were infiltrated with molten paraffin with an automatic tissue processor. 

### 2.2. Double-Labeling Immunohistochemistry

Archival formalin-fixed, paraffin-embedded primary human tumors of the central nervous system biopsies were sectioned into 4 μm thickness with a microtome and placed on charged microscope slides.

Sections were deparaffinized by heating slides in a tissue-drying oven for 1 h at 60 °C and then washing in fresh xylene two times for 10 min each at room temperature. Sections were rehydrated in a graded alcohol scale and rinsed in Tris-buffered saline solution (TBS) added with 0.025 Triton X-100 (TBS-Tr), and antigen retrieval was performed in 0.01 M sodium citrate pH 6.0 buffer for 20 min at 96–98 °C. Non-specific binding sites were blocked by Dual Endogenous Enzyme-Blocking target (S2003, Agilent Dako, Glostrup, Denmark) for 10 min.

Afterward, the sections were incubated with anti-Mitochondria (mouse, 1:400, MAB1273, Sigma-Aldrich, Merck KGaA, Darmstadt, Germany), anti-S100A10 (mouse, 1:50, 4E7E10, Novus Biologicals, Bio-Techne Ltd., Abingdon, U.K.), and anti-P-gp (mouse, 1:20, C494, Signet Laboratories, Dedham, MA, USA) primary antibodies diluted in antibody diluent (ab64211, Abcam, Cambridge, U.K.) for 30 min at room temperature. Rinsed in TBS-Tr, sections were incubated with Dako REAL™ Detection System, Alk Phos/RED, and Rb/M (K5005, Agilent Dako, Glostrup, Denmark) following the manufacture instructions to detect antibodies’ immunostaining signal in red.

To follow, slides were incubated with the other primary antibody to detect the second target. Briefly, sections were rinsed in TBS-Tr and treated with 3% H_2_O_2_ for 10 min. Slides were rinsed in TBS-Tr, and the Dual Endogenous Enzyme-Blocking target (S2003, Agilent Dako, Glostrup, Denmark) was applied for a second time. Afterward, the sections were incubated with anti-CD31 (rabbit, 1:60, ab28364, Abcam, Cambridge, U.K.) primary antibody diluted in antibody diluent (ab64211, Abcam, Cambridge, U.K.) for 30 min at room temperature. Rinsed in TBS-Tr, sections were incubated with biotinylated polymer and streptavidin-HRP for 10 min each (TP-060-HLX, Thermo Scientific, Fremont, CA, USA). The immunodetection was performed in distillate water with a DAB substrate kit for peroxidase (SK-4100, Vector Laboratories, Burlingame, CA, USA) for 2 min or with Vina Green (BRR807AH, Bio-Optica, Concord, CA, USA) for 10 min at room temperature.

Subsequently, the sections were counterstained with Gill no.1 hematoxylin (GHS132-1L, Sigma-Aldrich, Merck KGaA, Darmstadt, Germany) and mounted with Ecomount (EM897L, Biocare Medical, Prinsessegracht, The Netherlands). Negative controls were prepared by omitting the primary antibodies and mismatching the secondary antibodies.

### 2.3. Identification and Quantification of Vasculatures

A whole section was imaged from each biopsy using the scanning platform Aperio ScanScope CS (Leica Biosystems, Nussloch, Germany) at the maximum magnification available (40×).

Slides were viewed and analyzed remotely by two investigators on nine selected fields at 20× magnifications employing tools and algorithms embedded in the ImageScope v.11.2.0.780 (Leica Biosystems, Nussloch, Germany) [37]. The same layers were selected for the different immunolabeling, working with serial sections.

Any luminal structures positive for the expression of endothelial cell marker CD31 or markedly defined by P-gp- or S100A10- or mitochondria-positive cells and containing red blood cells or double-positive for CD31 and P-gp/S100A/mitochondria were considered blood vessels. 

All immunolabels alone or as colocalization have been quantified by Aperio colocalization algorithm using the double-labeling mode (all the relative markup layers are presented in Figures). The algorithm calculates the contribution of each stain at each pixel position in the image and determines the locations of individual proteins as well as the degree of “colocalization”, that is, whether the proteins appear alone or together. The colocalization algorithm classifies each pixel as either part of a single stain or representing a combination of stains based on the separated stains’ intensities. After the colocalization algorithm has been run, the software produces a markup image in which the brown chromogen-labeled area is visible in green, red chromogen in brown/red, green chromogen in green, and the colocalization in yellow.

### 2.4. Statistical Analysis

Data graphs are presented as means ± SD (standard deviation). The distribution of datasets was assessed using a D’Agostino and Pearson omnibus normality test. Two-way ANOVA with post hoc Tukey’s multiple comparisons test and Spearman nonparametric correlation analyses were performed using the GraphPad Prism software. Calculated *p*-value was summarized in Figure panels as * *p* < 0.05; ** *p* < 0.01; *** *p* < 0.001; **** *p* < 0.0001.

## 3. Results

### 3.1. P-gp/CD31 and Mitochondria/CD31 Expression and Colocalization in Central Nervous System Tumors

**Figure 2:**
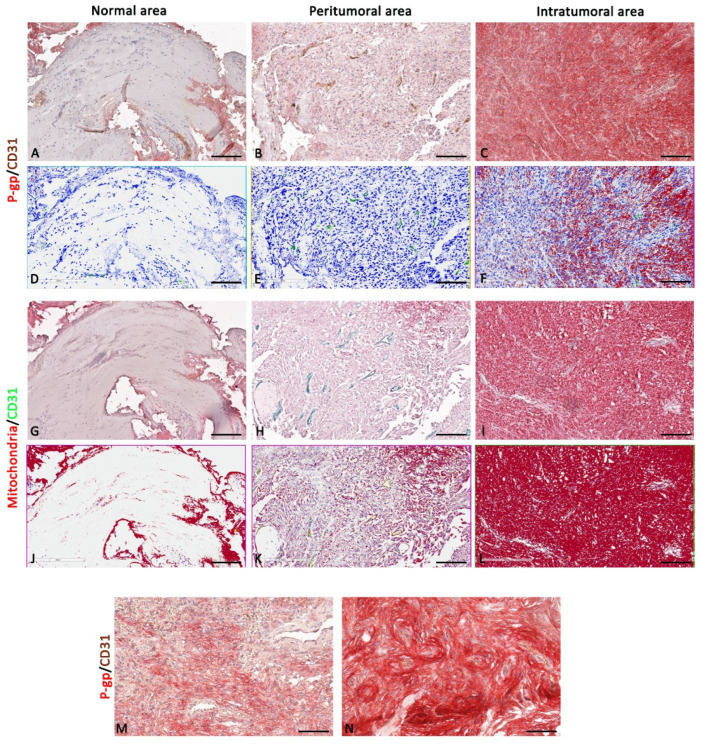
P-gp/CD31 and mitochondria/CD31 expression and colocalization in meningioma. P-gp, mitochondria, and CD31 were evaluated by immunohistochemistry within the same spatial ROIs on meningioma serial sections. P-gp (**A**–**F**) and mitochondria (**G**–**L**) expression progressively increase from normal to intratumoral areas. CD31 expression is reduced in the intratumoral areas compared with peritumoral and normal ones (**A**–**L**). Intratumoral areas show vascular co-option (**M**,**N**). Scale bar: (**A**–**L**) 100 μm; (**M**,**N**) 30 μm. Below each micrograph is the relative markup image generated by the Aperio colocalization algorithm.

**Figure 3:**
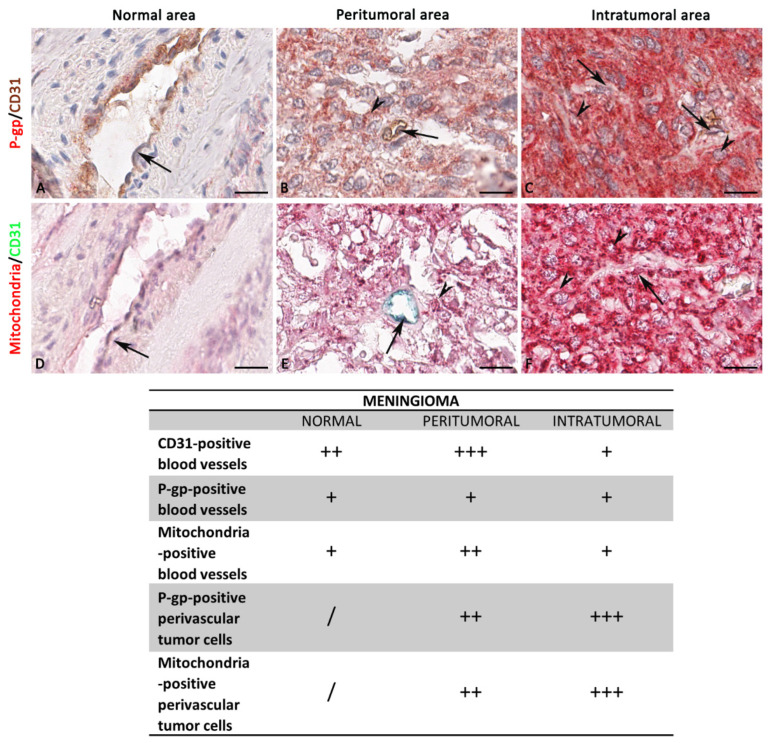
Focus on meningioma blood vessels and tumor cells. High magnification of P-gp, mitochondria, and CD31 immunohistochemistry reactions, within the same spatial ROIs on meningioma serial sections, and the summary table below show the differential expression of these markers by ECs and tumor cells. P-gp (**A**–**C**) and mitochondria (**D**–**F**) expression by tumor cells progressively increase from normal to intratumoral areas. Mitochondria + CD31-positive ECs show a higher proliferative profile in the peritumoral area (**E**), while there are no relevant differences in Pg-P expression. CD31 expression is reduced in the intratumoral areas (**C**,**F**) compared with the others. The table shows the semi-quantitative scoring of IHC reactivity: “/”, very weak or absent; “+”, weak; “++”, intermediate; “+++”, strong. Arrows: ECs; arrowheads: tumor cells. Scale bar: (**A**–**F**) 16 μm.

**Figure 4:**
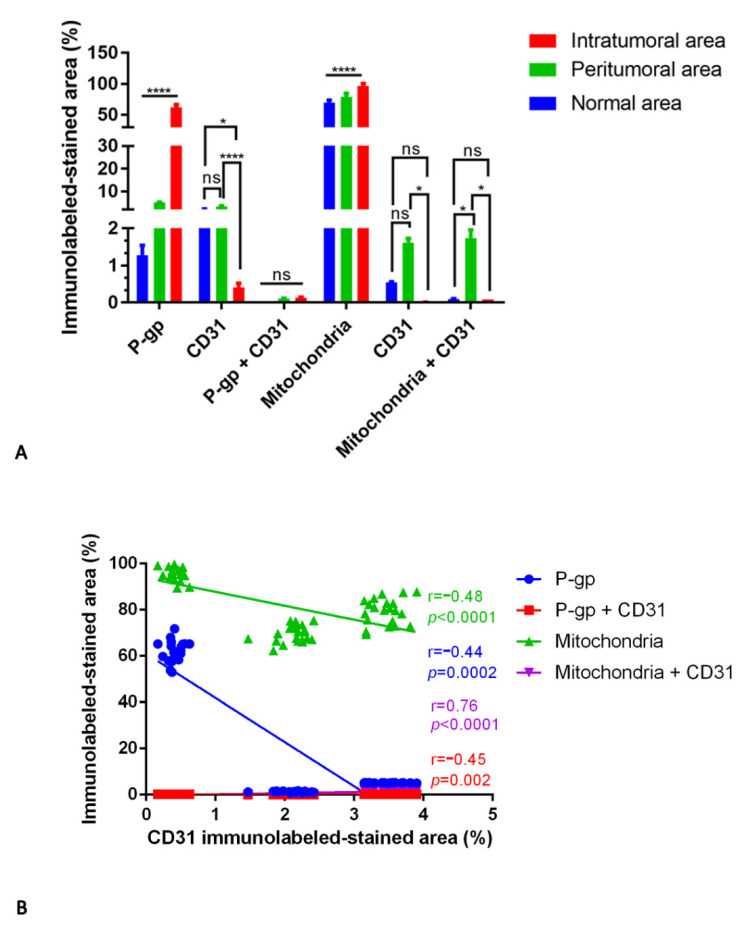
P-gp/CD31 and mitochondria/CD31 quantification in meningioma. P-gp, mitochondria and CD31 were evaluated by immunohistochemistry and were estimated as a percentage of digital quantification within the same spatial ROIs on meningioma serial sections using the colocalization algorithm. P-gp and mitochondria expression progressively increase from normal to intratumoral areas (**A**). CD31 expression is reduced in the intratumoral areas compared with peritumoral and normal ones (**A**). No significant differences are noted in the P-gp + CD31 (**A**) colocalized expression in all three areas. Significant is the mitochondria + CD31 (**A**) colocalized expression in the peritumoral area compared to the others. Linear regression analysis (**B**) shows a positive and very strong statistical correlation between the CD31 expression and the mitochondria + CD31 co-expression. The CD31 correlation with P-gp, P-gp + CD31, or mitochondria is fair [For P-gp: Y = −19.1*X + 60.99; for P-gp + CD31: Y = −0.01274*X + 0.1094; for mitochondria: Y = −6.037*X + 93.85; for mitochondria + CD31: Y = 0.5011*X − 0.3575]. Data are reported as mean ± SD, and the Tukey’s post hoc test was used to compare all groups after two-way ANOVA. N = 22. ns, not significative; * *p* < 0.05; **** *p* < 0.0001.

**Figure 5:**
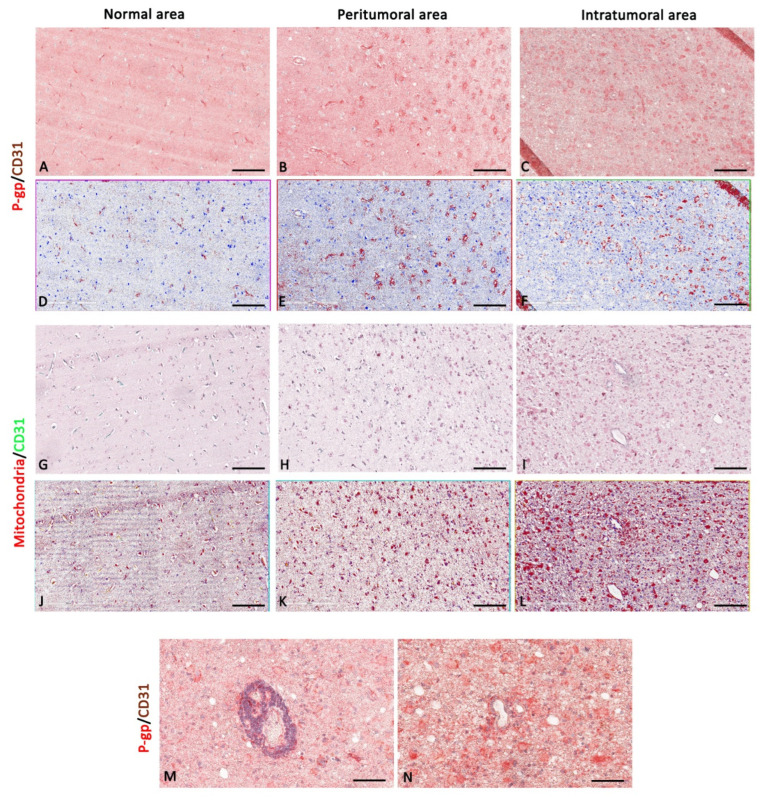
P-gp/CD31 and mitochondria/CD31 expression and colocalization in IDH-mutant astrocytoma. P-gp, mitochondria, and CD31 were evaluated by immunohistochemistry within the same spatial ROIs on IDH-mutant astrocytoma serial sections. P-gp (**A**–**F**) and mitochondria (**G**–**L**) expression progressively increase from normal to intra-tumoral areas. CD31 expression is not modified in the different areas (**A**–**L**). Intratumoral areas show perivascular satellitosis with lymphocytic infiltrate (**M**) or perivascular satellitosis solely (**N**). Scale bar: (**A**–**L**) 100 μm; (**M**,**N**) 30 μm. Below each micrograph is the relative markup image generated by the Aperio colocalization algorithm.

**Figure 6:**
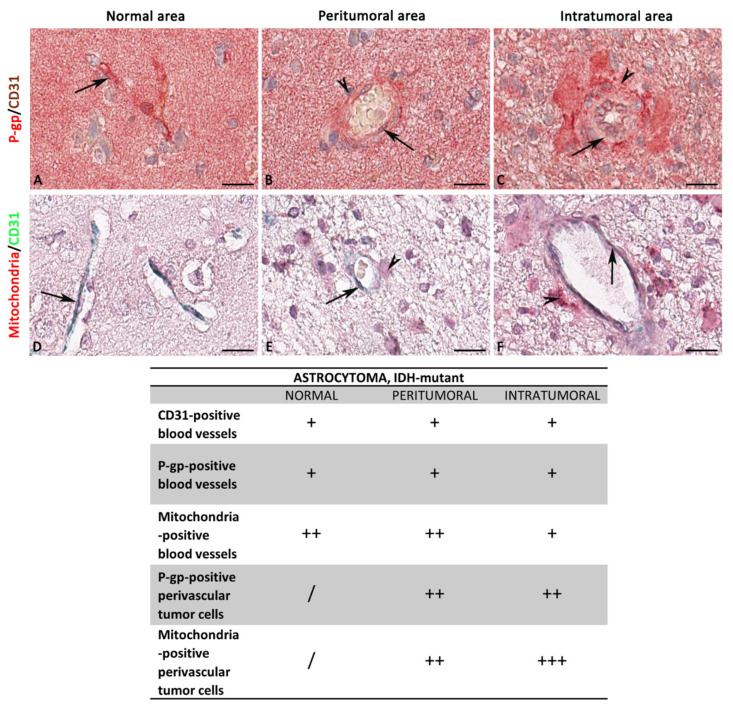
Focus on IDH-mutant astrocytoma blood vessels and tumor cells. High magnification of P-gp, mitochondria, and CD31 immunohistochemistry reactions, within the same spatial ROIs on IDH-mutant astrocytoma serial sections, and the summary table below show the differential expression of these markers by ECs and tumor cells. P-gp (**A**–**C**) and mitochondria (**D**–**F**) expression by tumor cells progressively increase from normal to intratumoral areas. Mitochondria + CD31-positive ECs show a reduced proliferative profile in the intratumoral area (**F**), while no relevant differences exist in P-gp expression by ECs. No relevant differences in CD31 expression also. The table shows the semi-quantitative scoring of IHC reactivity: “/”, very weak or absent; “+”, weak; “++”, intermediate; “+++”, strong. Arrows: ECs; arrowheads: tumor cells. Scale bar: (**A**–**F**) 16 μm.

**Figure 7:**
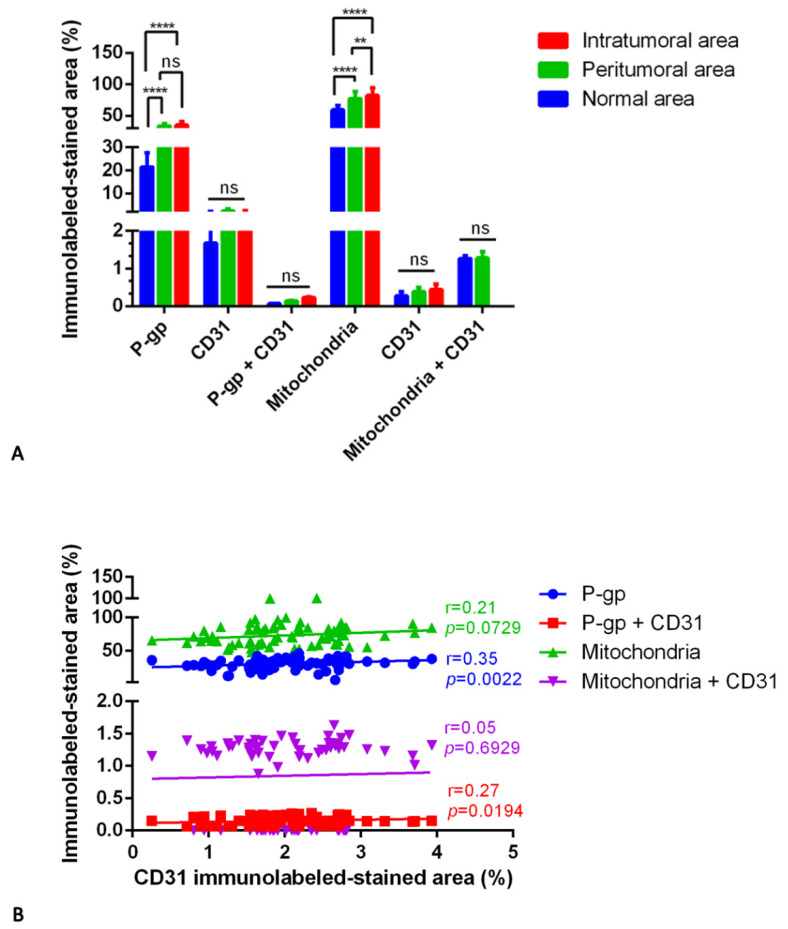
P-gp/CD31 and mitochondria/CD31 quantification in IDH-mutant astrocytoma. P-gp, mitochondria, and CD31 were evaluated by immunohistochemistry and were estimated as a percentage of digital quantification within the same spatial ROIs on IDH-mutant astrocytoma serial sections using the colocalization algorithm. P-gp and mitochondria are significantly overexpressed in peritumoral and intratumoral areas versus the normal one (**A**). No significant differences are noted in CD31, P-gp + CD31, or mitochondria + CD31 expression in all three areas (**A**). Linear regression analysis (**B**) shows a poor or fair statistical correlation between the CD31 expression and the other immunolabeled markers [For P-gp: Y = 2.824*X + 24.30; for P-gp + CD31: Y = 0.01772*X + 0.1134; for mitochondria: Y = 4.024*X + 64.79; for mitochondria + CD31: Y = 0.02627*X + 0.7950]. Data are reported as mean ± SD, and the Tukey’s post hoc test was used to compare all groups after two-way ANOVA. N = 25. ns, not significative; ** *p* < 0.01; **** *p* < 0.0001.

**Figure 8:**
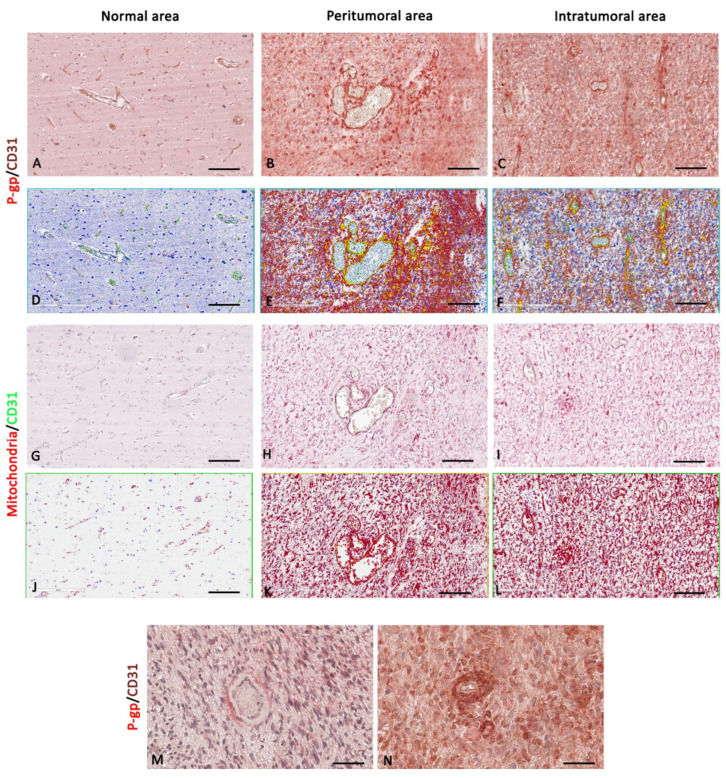
P-gp/CD31 and mitochondria/CD31 expression and colocalization in IDH-WT glioblastoma. P-gp, mitochondria, and CD31 were evaluated by immunohistochemistry within the same spatial ROIs on IDH-WT glioblastoma serial sections. P-gp (**A**–**F**), mitochondria (**G**–**L**), and CD31 (**A**–**L**) expression increase in peritumoral and intratumoral areas compared with the normal one. Peritumoral areas show vascular co-option as P-gp^+^ tumor cells surrounding ECs (**M**,**N**). Scale bar: (**A**–**L**) 100 μm; (**M**,**N**) 30 μm. Below each micrograph is the relative markup image generated by the Aperio colocalization algorithm.

**Figure 9:**
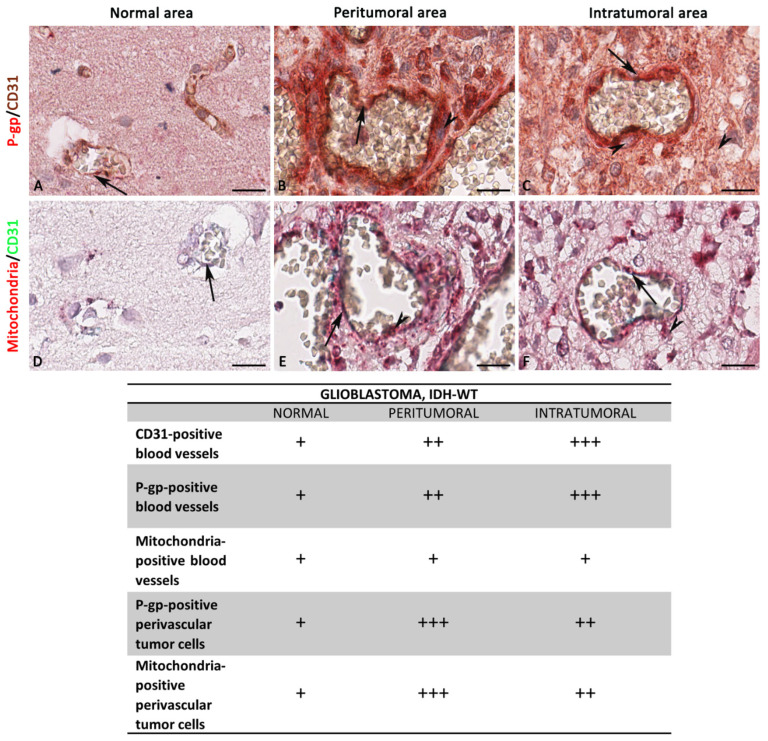
Focus on IDH-WT glioblastoma blood vessels and tumor cells. High magnification of P-gp, mitochondria, and CD31 immunohistochemistry reactions, within the same spatial ROIs on IDH-WT glioblastoma serial sections, and the summary table below show the differential expression of these markers by ECs and tumor cells. P-gp (**A**–**C**) and mitochondria (**D**–**F**) expression by tumor cells are increased in the peritumoral area compared with the others. CD31 and P-gp (**A**–**C**) expression by ECs are increased in the intratumoral area compared with the others. The table shows the semi-quantitative scoring of IHC reactivity: “+”, weak; “++”, intermediate; “+++”, strong. Arrows: ECs; arrowheads: tumor cells. Scale bar: (**A**–**F**) 16 μm.

**Figure 10:**
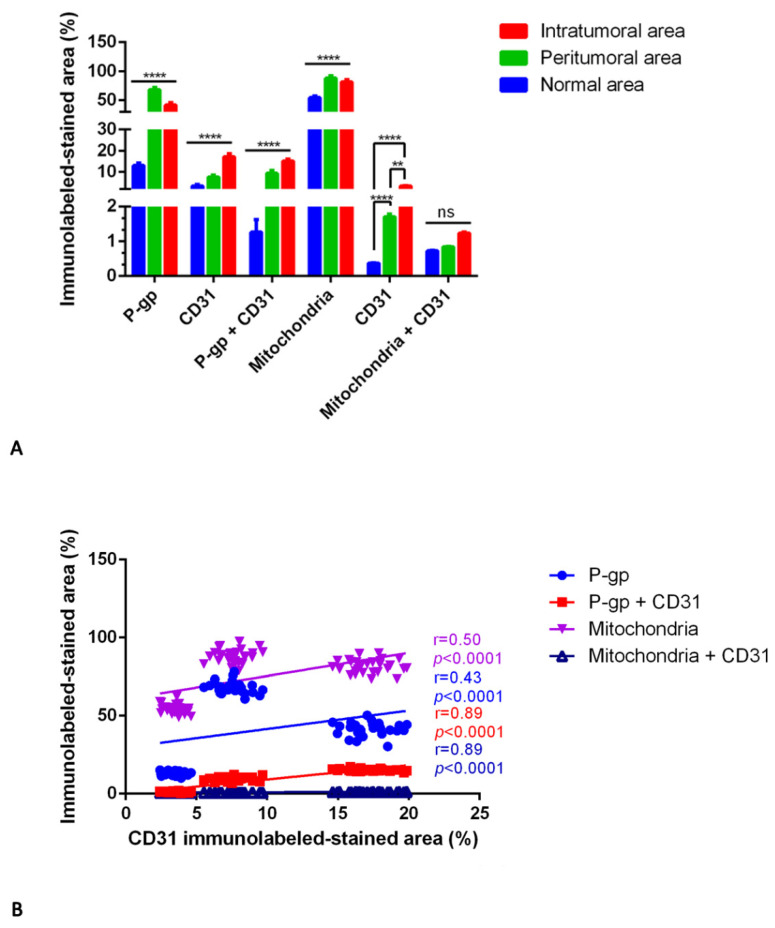
P-gp/CD31 and mitochondria/CD31 quantification in IDH-WT glioblastoma. P-gp, mitochondria and CD31 were evaluated by immunohistochemistry and were estimated as a percentage of digital quantification within the same spatial ROIs on IDH-WT glioblastoma serial sections using the colocalization algorithm. P-gp and mitochondria are significantly overexpressed in peritumoral areas versus the normal and intratumoral ones (**A**). CD31 and P-gp + CD31 progressively increased from normal to intratumoral areas. No significant differences are noted in mitochondria + CD31 expression in all three areas (**A**). Linear regression analysis (**B**) shows a positive and very strong statistical correlation between the CD31 expression and the P-gp + CD31 or mitochondria + CD31 co-expression (**B**). The CD31 correlation with P-gp or mitochondria is fair (**B**). [For P-gp: Y = 1.180*X + 29.77; for P-gp + CD31: Y = 0.9135*X + 0.02263; for mitochondria: Y = 1.481*X + 60.67; for mitochondria + CD31: Y = 0.03663*X + 0.5905]. Data are reported as mean ± SD, and the Tukey’s post hoc test was used to compare all groups after two-way ANOVA. N = 30. ns, not significative; ** *p* < 0.01; **** *p* < 0.0001.

The P-gp/CD31 and mitochondria/CD31 double immunostaining and the relative morphometric evaluation revealed a significant increased P-gp and mitochondria expression in perivascular tumor cells of co-opted blood vessels. These findings were observed both in the peritumoral and intratumoral areas of all three types of central nervous system tumors compared with the normal areas of the same sections (Figure 2, Figure 3, Figure 4, Figure 5, Figure 6, Figure 7, Figure 8, Figure 9 and Figure 10).

In more detail, in meningioma, a progressively increased and significant P-gp and mitochondria expression was noted from normal through peritumoral to intratumoral areas (Figure 2 and Figure 3).

The morphometric results for CD31 protein expression in ECs as brown or green chromogens were not perfectly congruent. However, in both immunostainings, the CD31 reduction is evident in the intratumoral areas compared with peritumoral and normal ones (Figure 4A). No significant differences have been identified in the P-gp/CD31 or mitochondria/CD31 colocalized expression in all three meningioma areas (Figure 4A). 

Blood vessel co-option was histologically observable in some meningioma areas, particularly the intratumoral ones, as perivascular satellitosis of tumor cells (Figure 2M,N). Positive and very strong was the statistical correlation between the CD31 expression and the mitochondria/CD31 co-expression (Figure 4B), while with the others, single or double immunolabeling was fair. Different markers’ expression in meningioma peritumoral and intratumoral areas at high magnification are summarized in the table of Figure 3.

In IDH-mutant astrocytoma, P-gp was overexpressed in both peri- and intratumoral areas compared with normal ones (Figure 5 and Figure 6).

The morphometric results for CD31 protein expression and its co-expression with P-gp or mitochondria were not statistically significant (Figure 7).

Blood vessel co-option was morphologically detectable in some astrocytoma areas, especially in the intratumoral ones, as perivascular infiltration of tumor cells and accompanying lymphocytes (Figure 5M) or perivascular satellitosis consisting of solely tumor cells (Figure 5N). Not significant or poor was the statistical correlation between the CD31 expression and the other single or double immunolabeling (Figure 7B). Different markers’ expression in astrocytoma peritumoral and intratumoral areas at high magnification is summarized in the table of Figure 6.

In IDH-WT glioblastoma, the expression of P-gp and mitochondria is strongly and significantly increased in the peritumoral areas compared with the intratumoral and normal areas (Figure 8, Figure 9 and Figure 10).

A progressively increased and significant CD31 expression and P-gp/CD31 co-expression were noted from normal through peritumoral to intratumoral areas (Figure 8 and Figure 9). No significant differences were identified in the mitochondria/CD31 colocalized expression in all three glioblastoma areas although an increased expression is evident in the intratumoral area (Figure 10). Vascular co-option was noted as perivascular satellitosis in some glioblastoma areas, particularly in the peritumoral ones (Figure 8M,N). Positive and very strong were the statistical correlations between the CD31 expression and the P-gp/CD31 or mitochondria/CD31 co0expression (Figure 10B), while with the single immunolabeling, they were fair. Different markers’ expression in IDH-WT glioblastoma peritumoral and intratumoral areas at high magnification is summarized in the table of Figure 9.

#### Summary Results

Overall, morphological and morphometric evaluations revealed in meningioma a progressively increased P-gp and mitochondria expression by tumor cells from the normal to the intratumoral area, a strongly reduced CD31 expression by ECs of the intratumoral area, and a higher mitochondria + CD31 expression by ECs of peritumoral area. These results indicated the presence of vascular co-option in the intratumoral area of meningioma and the sprouting angiogenesis in the peritumoral one.

For IDH-mutant astrocytoma, the results revealed an increased P-gp expression by tumor cells in the peritumoral and intratumoral areas compared with normal one, a progressively increased mitochondria expression by tumor cells from normal to the intratumoral area, and no significant data concerning CD31 and P-gp + CD31 by ECs in all three areas. Although mitochondria + CD31 expression by ECs results are not significant in all three areas, a reduced expression was appreciable in the intratumoral area. These results indicated the presence of vascular co-option in the intratumoral area of IDH-mutant astrocytoma.

For IDH-WT glioblastoma, the results revealed an increased P-gp and mitochondria expression by tumor cells in the peritumoral and intratumoral area compared with the normal one and a significantly higher expression in the peritumoral area compared with intratumoral one as well as a progressively increased CD31 and CD31 + mitochondria expression by ECs from the normal to the intratumoral area; although mitochondria + CD31 expression by ECs results is not significant in all three areas, an increased expression was appreciable in the intratumoral area. These results indicated the presence of vascular co-option in the peritumoral area of IDH-WT glioblastoma and confirmed the increased angiogenesis in the intratumoral area.

### 3.2. P-gp/CD31, Mitochondria/CD31, and S100A10/CD31 Expression and Colocalization in Glioblastoma

In addition to P-gp, mitochondria, and CD31, we explored the spatial and amount distribution of the S100A10 as a specific biomarker of glioma cells.

**Figure 11:**
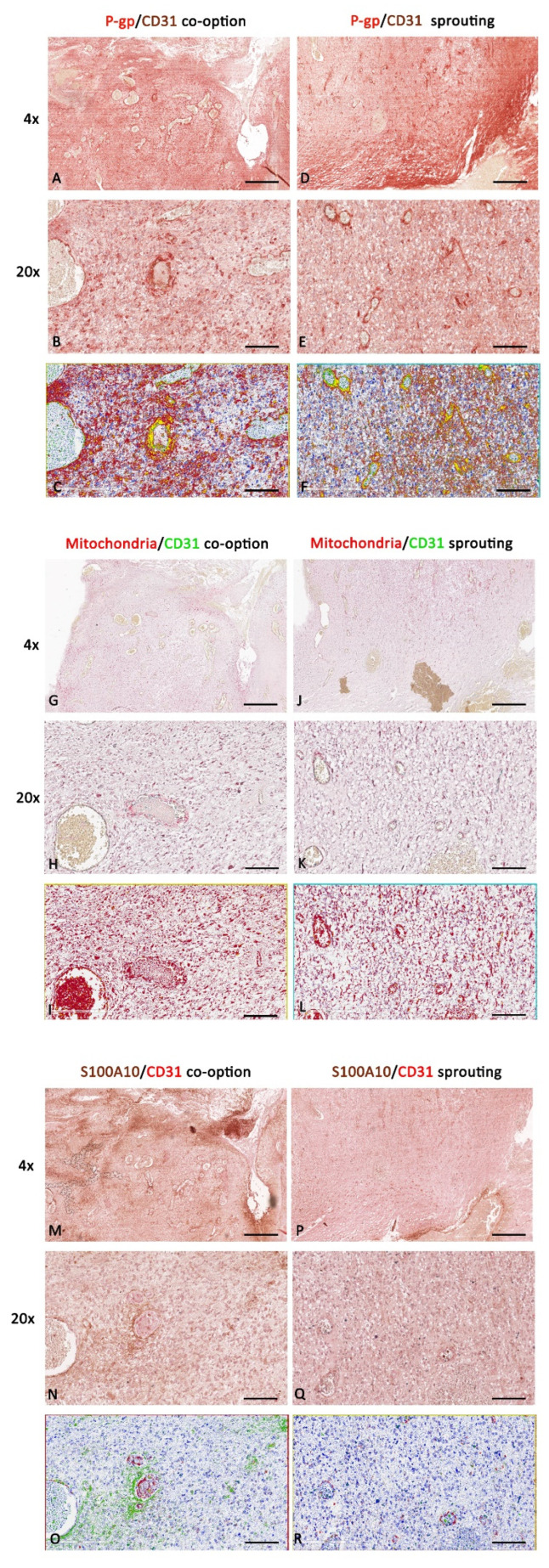
P-gp/CD31, mitochondria/CD31, and S100A10/CD31 expression and colocalization in IDH-WT glioblastoma. P-gp, mitochondria, S100A10, and CD31 were evaluated by immunohistochemistry within the same spatial ROIs on serial sections using the colocalization algorithm. P-gp, mitochondria, and S100A10 expression increase in the tumor area where vascular co-option prevails over sprouting angiogenesis (**A**–**C**,**G**–**I**,**M**–**O**). In the area where sprouting angiogenesis prevails, the CD31 expression (**D**–**F**,**J**–**L**,**P**–**R**) and the P-gp + CD31 co-expression (**D**–**F**) are increased compared with the area with a prevalence of vascular co-option (**A**–**C**). Scale bar: 4×, 300 μm; 20×, 100 μm. Below each micrograph is the relative markup image generated by the Aperio colocalization algorithm.

Based on the luminal or abluminal lining and placement of CD31^+^ endothelial and/or P-gp^+^/mitochondria^+^/S100A10^+^ tumor cells nucleus, blood vessels were identified and divided into two groups corresponding to co-opted vessels (formed by vascular co-option) or sprouted vessels (created by sprouting angiogenesis) (Figure 11). 

**Figure 12:**
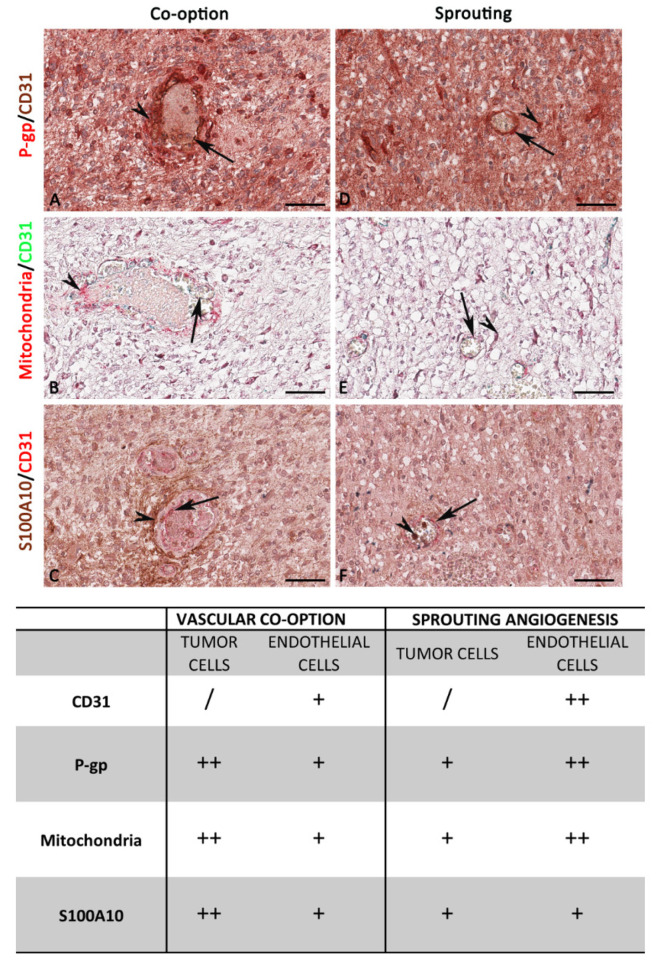
Focus on IDH-WT glioblastoma blood vessels and tumor cells. High magnification of P-gp, mitochondria, S100A10, and CD31 immunohistochemistry reactions, within the same spatial ROIs on IDH-WT glioblastoma serial sections, and the summary table below show the differential expression of these markers by ECs and tumor cells. In the area where vascular co-option occurs, tumor cells overexpressed P-gp (**A**), mitochondria (**B**), and S100A10 (**C**), while ECs do not reveal substantial differences. In the area where sprouting angiogenesis occurs, tumor cells do not reveal substantial differences, while ECs overexpressed P-gp (**D**) and mitochondria (**E**). The table shows the semi-quantitative scoring of IHC reactivity: “/”, very weak or absent; “+”, weak; “++”, intermediate. Arrows: ECs; arrowheads: tumor cells. Scale bar: (**A**–**F**) 16 μm.

The double-labeling immunohistochemistry and the relative morphometric evaluation revealed, in tumor cells, a significantly increased P-gp, mitochondria, and S100A10 expression in the area where vascular co-option occurs compared with the area of the same sections where the sprouting angiogenesis occurs (Figure 11). Moreover, in vascular co-option (Figure 11B,H,N and Figure 12) areas, the blood vessels, showing a reduced CD31 expression compared to sprouted vessels, appear surrounded by P-gp- or S100A10-positive tumor cells with a not-indifferent expression of mitochondria (Figure 11H and Figure 12), indicative of boosted metabolic activity in tumor cells. Different markers’ expression in IDH-WT glioblastoma during vascular co-option or sprouting angiogenesis at high magnification is summarized in the table of Figure 12.

**Figure 13:**
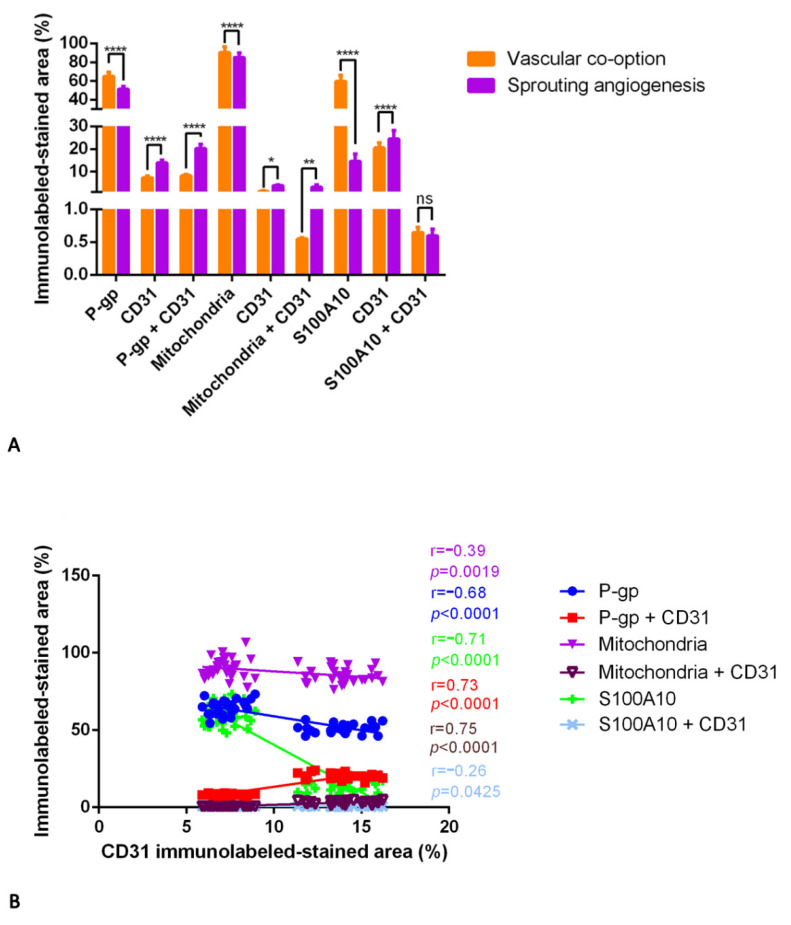
P-gp/CD31, mitochondria/CD31, and S100A10/CD31 quantification in IDH-WT glioblastoma. P-gp, mitochondria, S100A10, and CD31 were evaluated by immunohistochemistry and were estimated as a percentage of digital quantification within the same spatial ROIs on IDH-WT glioblastoma serial sections using the colocalization algorithm. P-gp, mitochondria, and S100A10 are significantly overexpressed where vascular co-option occurs versus sprouting angiogenesis (**A**). Where sprouting angiogenesis occurs, CD31, P-gp + CD31, and mitochondria + CD31 are overexpressed versus vascular co-option and progressively increased from normal to intratumoral areas. No significant differences are noted in S100A10 + CD31 expression (**A**). Linear regression analysis (**B**) shows a positive and very strong statistical correlation between the CD31 expression and mitochondria + CD31 co-expression. Moderate is the correlation of CD31 expression with P-gp or S100A10 expression or with P-gp + CD31 co-expression (**B**). Fair is the CD31 correlation with mitochondria or S100A10 + CD31 (**B**) [For P-gp: Y = −1.815*X + 77.15; for P-gp + CD31: Y = 1.636*X − 3.036; for mitochondria: Y = −0.7160*X + 95.28; for mitochondria + CD31: Y = 0.3668*X − 2.002; for S100A10: Y = −6.147*X + 102.0; for S100A10 + CD31: Y = −0.007386*X + 0.6995]. Data are reported as mean ± SD, and the Tukey’s post hoc test was used to compare all groups after two-way ANOVA. N = 30. ns, not significative; * *p* < 0.05; ** *p* < 0.01; **** *p* < 0.0001.

In contrast, in the areas of sprouting angiogenesis, the blood vessels appear with an increased CD31 expression, P-gp/CD31 (Figure 11E), and mitochondria/CD31 (Figure 11K) co-expression, which is indicative of ECs proliferation and migration (Figure 12). The differences in S100A10/CD31 co-expression were insignificant in the two methods of tumor growth (Figure 13A).

We did not quantify the peritumoral area in which there is vessel co-option with respect to the intratumoral one, but 28/30 glioblastomas showed co-opted blood vessels in the peritumoral area. On the contrary, 30/30 glioblastomas showed sprouting angiogenesis in the intratumoral area.

Positive and very strong was the statistical correlation between the CD31 expression and the mitochondria/CD31 co-expression (Figure 13 B). Moderate was the correlation between the CD31 expression and the P-gp or S100A10 expression or with P-gp/CD31 co-expression, while with the mitochondria expression and S100A10/CD31 co-expression, the correlations were fair.

Summary Results

Overall, these morphological and morphometric evaluations in IDH-WT glioblastoma revealed an increased P-gp, mitochondria, and S100A10 expression by tumor cells during vascular co-option and an increased CD31, P-gp + CD31, and mitochondria + CD31 expression by ECs during sprouting angiogenesis, and additionally, 93.33% of IDH-WT glioblastoma showed vascular co-option in the peritumoral area, while 100% of the same showed sprouting angiogenesis in the intratumoral area.

## 4. Discussion

In the present study, we explored the spatial and amount distribution of the P-gp as a drug efflux pump expressed on tumor and ECs plasma membranes [27], mitochondria as an indicator of increased cell activity and proliferation [40], S100A10 as a specific biomarker of glioma cells [33,34,41], and CD31 as ECs marker [42] across different brain tumors: human meningioma, IDH-mutant astrocytoma, and IDH-WT glioblastoma. Double immunolabeling reactions, with antibodies against the proteins mentioned above, were established to evaluate blood vessels in the same area of serial sections for the same bioptic specimens looking at normal, peritumoral, and intratumoral areas.

Our results have shown that vascular co-opted vessels could be recognized as metabolically overactive (evaluated as mitochondria expression) P-gp^+^ or S100A10^+^ tumor cells surrounding CD31^+^ endothelial cells with reduced P-gp and mitochondria expression. 

In vascular co-option, glioma cells, as single cells or as groups [43], migrate along and envelop pre-existing microvessels characterized by ECs expressing angiopoietin-2 (ANG-2) [44]. Subsequently, co-opted blood vessels undergo remodeling and collapse [45], resulting in loss of astrocyte-vascular coupling, blood–brain barrier breakdown, increased vessel leakage [46,47,48], and local hypoxia [49]. 

Angiogenesis, when occurring in gliomas, comes after the vessel co-option mechanism. The key angiogenic factor, vascular endothelial growth factor (VEGF), plays multiple roles and, in the presence of ANG-2, stimulates the migration and proliferation of ECs, inducing the sprouting of new blood vessels [50,51].

Intussusception is another neovascularization mechanism complementary to angiogenesis and characterized by the appearance of endothelial bridges within the pre-existing vascular network [52]. This process shows ECs-formed intraluminal pillars extending between opposite microvessels walls, with perivascular cells and extracellular matrix participating in their development [53].

Vasculogenesis has been recently proposed as an alternative neovascularization mechanism although with conflicting findings and not necessarily in chronological order after co-option and angiogenesis. This mechanism is based on the recruitment of endothelial progenitor cells (EPCs) deriving from bone marrow, which then differentiate into ECs to form new tumor vessels [54,55,56]. VEGF also plays an essential role in vasculogenesis by acting on VEGFR2-expressing EPCs, transforming into circulating endothelial progenitors (CEPs) that are incorporated into newly formed vessels [57,58]. In addition to EPCs, tumor-associated macrophages (TAMs) and circulating monocytes can differentiate into ECs, contributing to tumoral vasculogenesis [59]. 

Vascular mimicry represents another non-angiogenic method of tumor growth in which cancer cells form, by themselves, peculiar types of channels [60]. Vascular mimicry channels lack ECs, and their specific markers are PAS-positive and are formed by glioma cells with a stem-cell-like phenotype characterized by reduced glial fibrillary acidic protein (GFAP) and increased CD133 expression [14]. Moreover, these channels positively correlate with increased hypoxia-inducible factor-1 alpha (HIF-1α) expression, which drives the vascular mimicry process through the inner tumor mass [3]. A direct correlation has been demonstrated between vascular mimicry, tumor aggressiveness, and reduced survival [61,62,63]. Glioma cells can also transdifferentiate into ECs, as supported by the presence of the same genomic alteration in ECs and glioma cells, suggesting their possible neoplastic origin [61,62,64].

It was believed that targeting blood vessels would have been a winning strategy to counteract tumor progression, reducing blood supply and, consequently, tumor cell proliferation and survival. These therapeutics target specific angiogenic pathways, but cancer cells can find escape mechanisms, becoming even more aggressive in some cases [65,66,67]. Clinical studies have shown that not all malignancies responded favorably to AATs and even developed drug resistance [15]. AATs resistance can be intrinsic if present at the beginning of the treatment or acquired if it occurs after a transient therapy benefit, followed by relapse, i.e., a restart of tumor growth and progression [68]. Various cells, molecules, and biological processes play a role in AATs resistance: pro-angiogenic factors release, tumor cells extravasation and intravasation, metastatization, recruitment of tumor-associated microenvironment cells, and angiogenic or non-angiogenic methods of tumor growth [69]. 

Further investigation of all these neovascularization processes is fundamental to better understanding the mechanisms involved in glioma growth and progression and AATs resistance. Among the various neovascularization processes, vascular co-option may represent one of those principally responsible for the limited efficacy of AATs. In fact, glioma cells migrating along the abluminal surface of pre-existing microvessels satisfy their metabolic demands without stimulating neo-angiogenesis but continue to proliferate and invade surrounding normal tissue [70]. Overall, identifying all the actors involved in this mechanism may contribute to developing new and possibly more effective therapeutic strategies. Furthermore, for these reasons, it is vital to identify specific markers and techniques capable of distinguishing vascular co-option from other neovascularization mechanisms to understand its degree of involvement in different brain tumors and in different grades of the same histologic type.

In vascular co-option, ECs show a non-proliferating phenotype, while tumor cells are highly proliferative and metabolically overactive, generating energy through oxidative phosphorylation under normoxic or hypoxic conditions [40,71,72,73]. Based on these considerations, we demonstrated that vascular co-option mechanism could be reliably recognized, using immunohistochemical techniques, as metabolically overactive (evaluated as mitochondria expression) P-gp^+^ or S100A10^+^ tumor cells surrounding CD31^+^ ECs with reduced P-gp and mitochondria expression. Furthermore, we investigated differential CD31 and P-gp expression in normal, peritumoral, and intratumoral tissue, analyzing differences regarding the vascular co-option mechanism in three different human brain tumors: IDH-WT glioblastoma, IDH-mutant astrocytoma, and meningioma.

Overall, our morphological and morphometric evaluations across the different brain tumors revealed the presence of vascular co-option mainly in the intratumoral area of meningioma and astrocytoma and in the peritumoral area of glioblastoma and confirmed the increased angiogenesis in glioblastoma, particularly in the intratumoral areas, compared with the other two central nervous system tumors. Moreover, we demonstrated the dissimilar P-gp and P-gp/CD31 expression between co-opted and sprouted vessels and confirmed an essential method of glioblastoma cell growth and progression by vascular co-option in the peritumoral area [7].

These results may contribute to developing new and possibly more effective therapeutic strategies based on new markers for cellular components of the vessel wall and techniques that uncover the order and localization of vascularization mechanisms.

## Figures and Tables

**Table 1:** Patients characteristics.

Characteristics	IDH-WT Glioblastoma (n = 30)	IDH-Mutant Astrocytoma (n = 25)	Meningioma (n = 22)
**Gender**			
Male	18 (60%)	15 (60%)	6 (27.3%)
Female	12 (40%)	10 (40%)	16 (72.7%)
**Age group**			
<50	0	22 (88%)	5 (22.7%)
50–70	16 (53.3%)	3 (12%)	14 (63.7%)
>70	14 (46.7%)	0	3 (13.6%)
**Tumor location**			
Frontal lobe	13 (43.4%)	9 (36%)	5 (22.7%)
Parietal lobe	10 (33.3%)	11 (44%)	7 (31.8%)
Temporal lobe	6 (20%)	4 (16%)	4 (18.2%)
Occipital lobe	1 (3.3%)	1 (4%)	0
Cerebellum	0	0	0
Brainstem	0	0	0
Spinal cord	0	0	6 (27.3%)
**Grading**			
1	/	0	22 (100%)
2	/	21 (84%)	0
3	/	4 (16%)	0
4	30 (100%)	0	0

## Data Availability

The authors confirm that the data supporting the findings of this study are available within the article.
